# Microbial Air Quality in Neighborhoods near Landfill Sites: Implications for Public Health

**DOI:** 10.1155/2020/4609164

**Published:** 2020-07-11

**Authors:** Stephen T. Odonkor, Tahiru Mahami

**Affiliations:** ^1^School of Public Services and Governance, Ghana Institute of Management and Public Administration, Accra, Ghana; ^2^Biotechnology and Nuclear Agriculture Research Institute, Ghana Atomic Energy Commission, Accra, Ghana

## Abstract

Air pollution has been a major challenge worldwide particularly in the developing world. Improper waste disposal and management may result in microbial air pollution. In advanced countries, landfill sites are far from neighborhoods; however, the opposite is observed for landfill sites in the developing world. In Accra, some landfill sites are 100 meters from neighborhoods. The aim of this study was to assess the microbial air quality and associated environmental health hazards of landfill sites in selected districts in the Greater Accra Region of Ghana. A random sampling method was employed to select sampling sites across the dry and wet seasons from landfills and their corresponding neighborhoods. Results obtained showed a higher total count (CFU/m^3^) of bacteria and fungi in the air at the landfill sites than neighborhoods. Statistically significant variation (*p* < 0.05) in bacterial and fungal concentrations over two seasons was found for both landfills and neighborhoods. However, bacterial concentrations were significantly higher than fungal concentrations (*p* < 0.05) across seasons for all locations. *Staphylococcus epidermidis* was the highest (15.6 %) occurring microbe at both landfill sites and neighborhoods. This was followed by *Staphylococcus aureus* (12.7%). Other bacteria and fungi of public health importance such as *Pseudomonas aeruginosa*, *Escherichia coli*, *Aspergillus flavus*, and *Aspergillus niger* were also isolated from the study sites, above the WHO recommended levels. In conclusion, the landfill waste disposal and its close proximity to neighborhoods as observed in this study pose a potential environmental health risk, with dire implications for public health and safety. The government must enact and implement policies to regulate waste management and to ensure public safety.

## 1. Introduction

Air pollution has become an issue of great concern because of its impact on the health of the people [1]. Air pollution could cause serious respiratory complications [2]. The World Health Organization (WHO) reported in 2018 [3] that an estimated number of 3.8 million people die every year because of household air pollution and 9 out of 10 people breathe in poor-quality air daily. Control of air pollution has been a challenge over the years because of how challenging it is, to observe and determine the pollutants [4].

Most often air pollutants that are usually perceived to be associated with air pollution are particulate matter and greenhouses gases. The atmosphere contains a mixture of gases that make it an uncomfortable habitat for microbes because it is unable to provide the needed nutrients and physical conditions such as favorable oxygen levels for their growth [5]. However, a previous study by Smets et al. [6] suggested air could harbor bacterial and fungal communities. Microbial particles such as single spores, spores in aggregate form, pollens, bacterial cell, viral particles, mycelium, fungal spores, and other biological materials could be distributed in the air [5]. These microbial particles are known as bioaerosols, and their presence in the air significantly affects its quality [7]. Poor air quality is associated with increased risks of heart-related diseases [8].

The concentration or counts of microbes and their biological particles in the atmosphere are significantly influenced by the source of emission. The findings of Odeyemi et al. [9] in their study on bacteriological, mineral, and radioactive contents of leachate samples from the dumpsite of Ekiti State, Nigeria, indicate landfill sites could potentially harbor high concentrations of pathogenic microbes. Landfills refer to places or areas that harbor solid waste from different sources including municipal and industrial sources [10]. In Ghana and in most developing countries, towns in urban settings are untidy, with garbage, food chunks, polythene bags, cans, and quite unpleasantly human and animal feces accumulating at landfills which are in close proximity of up to 100 m near surrounding homes. Dumpsites of this nature are usually irksome ornamentally and produce irritating odor especially when there is the decomposition of organic matter present by bacteria [11]. Refuse dumps release bioaerosols in the air that are associated with pathogens known for causing fatal diseases like cholera and diarrhea [12]. It is of much concern when bioaerosols of this nature are present in the outdoor environment because it can be distributed over wide areas through various mechanisms into the indoor setting as well. Anaerobic microbes can convert organic matter in wastes into gases that are able to react with other compounds as they move through the air space to cause explosions, which are detrimental to human health [13]. Małecka-Adamowicz et al. [5] report that high concentration of microbes as well as low concentrations of specific microbes in the atmosphere can produce allergen. Allergens produced by fungi are associated with respiratory complications [14].

Furthermore, the habit of burning waste at landfills as a means of waste management is a common practice in Ghana, even in some areas in the regional capital. Burning activities can significantly contribute to air pollution [15]. Apart from the emission of greenhouse gases and other particulate matter, burning of waste at landfills could also emit aerosols from primary biological origins [16], subsequently leading to microbial air pollution. To minimize air pollution, sanitary landfills have been adopted by most developed countries for some time now [17]. Though the use of engineered landfills is increasingly becoming a common practice in developing countries [18], its adoption in Ghana is inefficient. There is insufficient information on the characteristics of these landfills which is very important in proper waste management at these sites [19]. As such, most landfills' operations in this country are far below the recommended standards of sanitary procedures [20]. According to Thompson [21], the scale of the landfills in Ghana is not large enough due to people living in their surroundings. Therefore, such persons are at risk of inhaling poor-quality air, possibly contaminated with pathogenic microbial bioaerosols.

It is therefore of great importance to assess air quality in vicinities or towns close to dumpsites, to constantly ascertain biological and physical health risks, to inform stakeholders, policymakers, and general populace. The aim of the study was to assess microbial air quality and its public health implication of neighborhoods around landfill sites in Accra Ghana.

## 2. Methodology

### 2.1. Description of the Study Location

**Figure 1:**
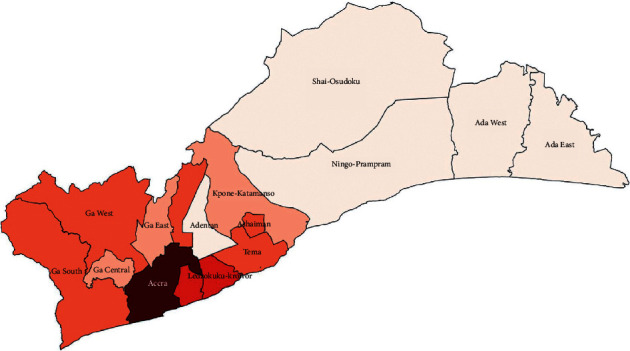
Map of Greater Accra Region.

The study was conducted in the Greater Accra Region of Ghana (Figure 1), which lies in the southeastern part of the country. The region occupies a total land area of 3,245 sq. km. It is the national capital of the 16 political regions in Ghana. It has a population density of 1,235.8 people per sq. km. The region is 90.5% urban with an annual urban growth rate of 3.1%. It experiences more inflows of people from other parts of the country than people moving out of the region [22].

The Greater Accra Region is the most urbanized region in the country with 87.4% of its total population living in urban centers. The region had a population of 4,010,054. Accra which is the regional as well as the national capital of Ghana accounts for over 2 million of the total number of inhabitants' resident in the region. Although Accra provides full waste collection services in parts of the city, waste is not collected from more than half of the city's households. Waste is collected by private companies that are regulated by the city. Accra generates nearly 900,000 metric tons of solid waste per year, with about 67% being organic matter. However, Accra does not have a formal organic waste diversion program, but two private companies are currently operating composting and recycling facilities. Accra's dumps have all been closed, and the city currently does not have its own solid waste disposal site. The collected waste is trucked to a landfill in a municipality approximately 37 kilometers away [22, 23].

### 2.2. Sampling Sites, Duration, and Frequency

The study was carried out at landfill/dumpsites and their corresponding homes in neighborhoods surrounding them. These were carried out in six (6) towns in selected districts in the Greater Accra Region of Ghana. The six sampling sites were labeled A through to F. Sampling of bacterial and fungal load in air samples was carried out covering both the dry and wet seasons throughout the year. Air samples were collected from selected dumpsites which were in proximity of about 50–150 m to the nearest neighborhoods. Indoor air samples were also collected from homes within the same study locations. Additionally, samples were collected from a further 2 km away from the dumpsites (used as control) for comparison with samples collected from neighborhoods in close proximities to the landfill sites.

### 2.3. Air Sampling and Collection Procedure

Microbial air samples were taken thrice a week at peak hours (10 : 00 am–8:00 pm in the dry season and 7 : 00 am–9 : 00 pm in the wet season) for each location. Sampling height was maintained at 1.5 m above ground level to signify the breathing zone. Air was sampled using a MAS-100 air sampling device with a flow rate of 100 litres per minute. Viable air sampling was achieved by this device through real-time regulation of air samples aspirated through the perforated lids via its advanced mass flow sensor. Samples collected were exerted on 90 mm Petri dishes for microbial enumerations. A large volume of 200–300 litres of air samples was collected from each of the sampling sites and locations at the selected districts in the study due to the general likelihood of having a low microbial population in the atmosphere.

### 2.4. Bacterial and Fungal Counts

Bacterial and fungal population was enumerated using plate count agar (PCA) incorporated with antifungal agent. The growth of bacteria was enumerated on PCA and that of fungi was cultured on dextrose agar. After culturing, bacterial plates were incubated at 37°C for up to 48 hours and fungi plates were incubated at 25°C–28°C for up to 6 days, and both tests were monitored daily. Average counts of colonies were enumerated and calculated as colony-forming units per cubic meter (CFU/m^3^) using the following formula:

(1) $$\begin{array}{c} \text{total counts}\left(\frac{\mathrm{cfu}}{m^{3}}\right)=\underset{\text{volume of air sampled}}{\left[\underline{\text{total colonies }\times 1000}\right]}. \end{array}$$

To have a more reliable data, microbial counts were corrected using Feller's [24] conversion formula:

(2) $$\begin{array}{c} \mathrm{Pr}=N\left[\frac{1}{N}+\frac{1}{N-1}+\frac{1}{N-2}+\cdots +\frac{1}{N-r+1}\right], \end{array}$$

where *N* = 400 (number of perforated holes on the lid of the air sampling device), *r* = number of coliform forming units counted on the specimen plates, and Pr = corrected total count of bacteria/fungi colonies in tested air samples.

### 2.5. Isolation and Identification of Bacteria and Fungi

Colonies of bacteria obtained on the PCA were first of all classified based on colonial morphological features. Staining, microscopy, and biochemical testing were used to identify the colonies. Analytical profile index (API) [25] was used to confirm the identities of bacteria isolates. Similarly, airborne fungal spores grown on the potato dextrose agar plates were grouped based on their colonial morphology. Identifying the genus of dominant fungal colonies was carried out using slides wetted with lactophenol blue which were then observed and identified under a microscope (400x).

### 2.6. Data Analysis

Data were analyzed using the Statistical Program for Social Sciences (SPSS) software. *χ*^2^ test was used to test the significant difference between microbial loads in the air samples at the dumpsites and their respective closest neighborhood. For normality, data obtained were checked using Kolmogorov–Smirnov's test. General parameters such as mean and standard deviation of plate counts (bacteria and fungi count) were established. Test of significance in microbial counts at the dumpsites and the neighborhoods was determined by *t*-test. The significance level was *α* = 5%.

## 3. Results

**Figure 2:**
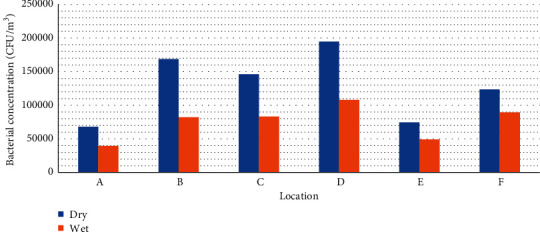
Seasonal variation of combined airborne bacterial counts at dumpsites and indoors per each location.

**Figure 3:**
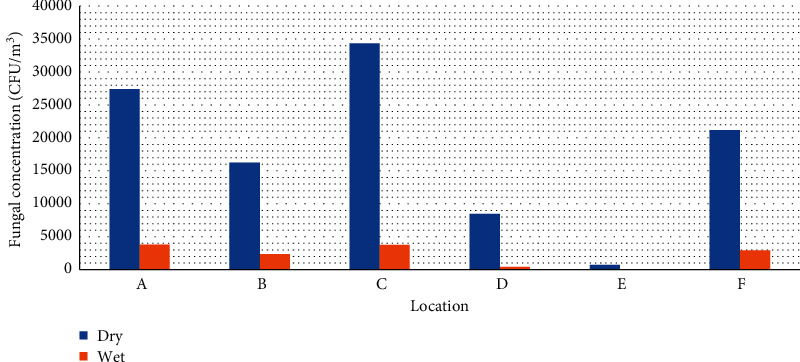
Seasonal variation of combined airborne fungal counts at dumpsites and indoors per each location.

The total measured bacterial and fungal load in air samples taken at the dumpsites and indoors at each location during the dry season was higher than that during the wet season as shown in Figures 2 and 3.

As shown in Table 1, the count of bacterial bioaerosols ranged from 108 CFU/m^3^ to 703 CFU/m^3^ across all six locations with a mean count of 407 CFU/m^3^. Fungal concentration ranged from 1 CFU/m^3^ to 200 CFU/m^3^ across the locations with a mean of 79.2 CFU/m^3^. Specifically, samples from dumpsite location D had the closest range of bacterial counts whilst location C had the closest range of fungal load. Additionally, data in Table 1 clearly indicate that the total load of bacteria in air samples collected at dumpsites was higher than that of fungi.

Indoor air samples collected from homes near dumpsites (about 50 to 150 m in proximity) had bacterial counts ranging from 30 CFU/m^3^ to 389 CFU/m^3^ and 0 CFU/m^3^ to 28 CFU/m^3^ for fungi with an overall mean count of 159.50 CFU/m^3^ and 9.76 CFU/m^3^, respectively (Table 2). Similarly, data in Table 2 indicate that bacterial load in air samples from homes was higher than that of fungi.

To meet the objectives of this study, it was necessary to specifically determine whether the landfills actually had a significant impact on airspace within their nearest corresponding neighborhoods. Therefore, indoor air samples were collected at homes across these same six study locations but a further 2 km away from the dumpsites/landfills (used as control) for comparison with samples collected from neighborhoods in close proximities to the landfill sites. In reference to Table 3, bacterial and fungal counts across homes in the study locations 2 km away from the landfills ranged from 1 CFU/m^3^ to 16 CFU/m^3^ and 1 CFU/m^3^ to 5 CFU/m^3^, with mean counts of 4.80 CFU/m^3^ and 0.93 CFU/m^3^, respectively.

Comparatively, the mean counts of microbial cells at dumpsites and nearest homes suggested microbial bioaerosol loads were significantly higher in samples from dumpsites than in samples collected indoors. To further test for significance in detail between the two categories of data, the *t*-test result revealed significant differences between some sets of the categorical data derived from the study. There were significant differences between fungal and bacterial population in dumpsite air samples and indoor air samples in the dry season (*p* < 0.005). In the wet season, though there was a highly significant difference (*p*=0.027) between bacteria cell counts in samples from dumpsites and those from homes, the difference of fungi population among these two study locations was insignificant (*p*=0.102).

The total number of bacteria and fungi species identified and isolated in the study is twelve and nine, respectively.  Table 4 represents the number of different species of bacteria isolated in the study from homes and dumpsites across both seasons. The total count of bacteria isolated was log_10_ (4.092). For each particular species, there were more bacteria isolated in the dry season than in the wet season. *Staphylococcus epidermidis* was the highest (15.6%) occurring bacteria. *Enterococcus faecalis* was the least (5.6%) occurring microbe.

Table 5 shows the different species of fungi isolated and their respective occurrences from homes and dumpsites across both seasons. The total number of fungi cells isolated was log_10_ (2.780). *Cladosporium spp* was the highest (17.8%) occurring fungi across seasons whilst *Penicillium oxalicum* was the least (4.5%) occurring fungi species. The total number of occurring bacteria in this study was about 21 times higher than that of fungi.

## 4. Discussion

The objective of this paper was to assess microbial air quality and its public health implication of neighborhoods around landfill sites in Accra, Ghana. Apart from other pollutants, examining microbial load in the atmosphere plays a key role in air quality assessment. Findings of microbial bioaerosol population in indoor air can help to project health dangers and to standardize indoor air quality control [26]. WHO's guideline for indoor air quality in 2009 revealed that indoor microbial pollutants usually originate from the outdoor environment which includes dumpsites [27].

Bacterial counts were higher during the dry season than during the wet season. This finding agreed with a similar study by Wemedo et al. [28]. In their study on the interaction between building design and indoor airborne microbial load, Wemedo et al. [28] established that airborne microbial population including bacteria was higher in the dry season than in the wet season with one of the major factors attributed to poor ventilation during the dry season as compared to the wet season. This is further espoused by Lis et al. [29], who revealed that ventilation which is needed in adequate levels in distribution or dispersion of bacterial spores across long distance to prevent their accumulation in the atmosphere is poor during the dry season. In Ghana and majority of the West African region, wind speed is low, temperatures are elevated, and humidity is low for greater periods of each day during the dry season, leading to poor distribution of air across the affected countries [30]. Therefore, the atmosphere at dumpsites and surrounding vicinities will tend to harbor a high number of bacteria spores in the dry season.

Fungal counts were equally higher during the dry season than during the wet season. Fungal growth is stimulated by wet conditions [31]. Though it could be expected that fungal counts will be higher in the wet season due to rainfall which stimulates their growth, it is not entirely accurate. This is because usually the biological agents of fungi that are a component of bioaerosols are spores. These spores are released from the growth sources and distributed effectively under high temperatures [32]. Spores of fungi belonging to the division, for example, Ascomycota tend to release spores under moist conditions. In Ghana, the atmospheric temperature is low at night, coupled with good aeration during the dry season which provides moist conditions for effective spores' distribution from fungi of that division. Moreover, a dry period immediately following a rainy season will spike the release of fungal spores into the atmosphere [33]. Thus, fungal spores are multiplied during the rainy season and are better released into the atmosphere during the dry periods. These explanations may be the reasons behind the higher bacterial and fungal counts observed in the dry season than in the wet season.

Data from Tables 1–3 showed that bacterial and fungal counts were higher in air samples collected from dumpsites than in samples from nearby vicinities and neighborhoods 2 km away from the dumpsites. Dumpsites have a considerable level of microbial contamination [34] because whenever waste is dumped on land, soil microbes especially anaerobic fungi and bacteria inhabit the waste and extract nutrients by carrying out decomposition [35]. Microbial population tends to multiply at the dumpsites/landfill sites and these dumpsites act as a source of microbiological agents (bioaerosols) in the atmosphere [36]. The soil is not the only source of microbes in dumpsites. Wastes like feces from both animals and humans contain loads of bacteria and fungi cells already. Thus, in the study, samples from the dumpsites had higher microbial counts as opposed to their respective neighborhoods nearby irrespective of the season. Nevertheless, the dumpsites contributed significantly to the bioaerosol population in the nearby vicinities. This is because the control air samples collected from neighborhoods that were 2 km away from the landfills across the locations had significantly lesser microbial counts (Table 3) than those from nearby locations (Table 2). According to Burkowska et al. [34], dumpsites could have a negative impact on the atmosphere in surroundings from few hundreds of meters up to one kilometer apart even if they are well protected. In a similar study by Odeyemi [9], the author found out that microbial loads in the air decreased further away from the dumpsites and this occurrence was attributed to the antimicrobial action of UV rays from sunlight that reduces atmospheric nutrients available for microbial use. The same reasons could explain similar observation in this study.

Among the isolated and identified species of bacteria observed in the study, the genera *Staphylococcus* which had the highest counts across the wet and dry seasons at the 6 locations is linked with multiple diseases including skin infections. In the UK, for instance, the species *Staphylococcus aureus* had been involved in increase in skin infections among children from 1997 to 2006 [37]. *S. epidermidis* labeled as an “accidental” pathogen has also been implicated in nosocomial infections in recent years [38]. *Enterococcus faecalis* which was also identified in the study could also cause infections, especially infections of the urinary tract, which are challenging to treat. This is further compounded as a result of bacteria resistance to a variety of antibiotics [39]. *Pseudomonas aeruginosa* which had the third highest overall occurrence count is a well-known pathogen that causes diseases such as the deadly bloodstream infection (BSI) disease [40]. In reference to Table 4, the ratio of occurrence between the dumpsites and homes of *Pseudomonas aeruginosa* was close across all the locations, thereby suggesting it may have established itself in the atmosphere of the communities. Some other species that were isolated such as E. *coli* and *Proteus spp* are also of clinical importance.

The genus of fungi mostly identified in the study is *Aspergillus*. *Aspergillus spp* is known for causing serious respiratory problems and invasive infections such as chronic necrotizing pneumonia and invasive pulmonary aspergillosis [41]. *Phanerochaete chrysosporium* and *Cladosporium spp* which were also identified are pathogens of serious clinical importance and are known to cause diseases such as granulomatous lung diseases and cerebral/cutaneous phaehyphomycoses [42].

The American Conference of Governmental Industrial Hygienists (ACGIH) standard microbial levels for bacteria in the atmosphere is 100 CFU/m^3^. For each of the study locations, the counts of total bacteria load in indoor air samples alone far exceeded the limit irrespective of the season, which have serious health repercussions. According to Ghosh and Srivastava [4], terrorist attacks using biological agents and the flu pandemonium in 2009 are important highlights calling for the need to carry out more research studies regarding the population of bioaerosols in the atmosphere especially in the indoor environment. Furthermore, the authors revealed that numerous diseases have been linked with poor air quality caused by bioaerosols with tuberculosis and severe acute respiratory syndrome (SARS) wreaking the greatest havoc socioeconomically. For fungi, WHO estimates a limit of 500 CFU/m^3^ which is higher than that for bacteria, since most infectious diseases are not associated with them. The fungi count from this study in some locations was far below the limit, during the wet and dry seasons. This observation could be as a result of low concentration of the fungi, as well as unfavorable environment conditions which might have impeded the propagation of the fungi in the air.

## 5. Conclusion

This study showed that dumpsites had an impact on the microbial air load and air quality in surrounding neighborhoods. Microbial loads in air samples were higher in the dry season than in the wet season. Though microbial population decreased in indoor setting as compared to the dumpsites, their bacterial microbial counts surpassed the acceptable limits. Bacteria is linked with hundreds of infectious human diseases. So, the findings of the study suggest that people living in these are prone to health threats because the air they inhale daily is of poor quality. Hence, it is recommended that dumpsites are located at least 2 km from neighborhood to avoid air contamination with pathogenic microbes.

Furthermore, almost every species of microbes isolated in the present study (especially those belonging to the bacteria domain) are capable of causing serious diseases that could subsequently lead to deaths of people they infect. In addition to allowing reasonable distances between the dumpsites and nearby towns, there should be sufficient ventilation and proper sanitation at homes to ensure microbial biological particles associated with bioaerosols are not concentrated at regions within the indoor airspace.

Finally, Ghana should direct its efforts towards the effective treatment of waste before disposal to reduce the population of microbes in them.

## Figures and Tables

**Table 1:** Descriptive statistics of bacterial and fungal concentrations at the landfill sites.

Location/site	Bacteria (ACGIH limit 100 CFU/m^3^)				Fungi (WHO limit 500 CFU/m^3^)			
	Mean ± SE	Min	Max	SD	Mean ± SE	Min	Max	SD
A	222.47 ± 4.12	120.	319	62.39	120.15 ± 2.71	90	200	40.98
B	525.21 ± 10.81	300	703	163.35	71.18 ± 1.62	23	90	24.47
C	412.99 ± 7.94	300	611	119.95	150.57 ± 1.97	120	200	29.77
D	631.41 ± 4.78	508	700	72.20	37.14 ± 2.25	6	99	34.10
E	235.14 ± 5.13	108	332	77.46	3.22 ± 0.11	1	6	1.71
F	418.38 ± 8.17	232	548	123.46	92.86 ± 4.45	30	200	67.14

**Table 2:** Descriptive statistics of bacterial and fungal concentrations at homes (indoor) near landfill sites.

Location/site	Bacteria (ACGIH limit 100 CFU/m^3^)				Fungi (WHO limit 500 CFU/m^3^)			
	Mean ± SE	Min	Max	SD	Mean ± SE	Min	Max	SD
A	76.27 ± 1.94	30	113	29.31	16.61 ± 0.31	10	22	4.68
B	215.14 ± 6.19	100	340	93.49	10.41 ± 0.08	9	12	1.35
C	227.94 ± 5.77	120	389	87.16	16.46 ± 0.26	12	23	3.93
D	222.47 ± 4.13	120	319	62.39	1.81 ± 0.06	1	3	0.98
E	91.22 ± 2.32	50	150	34.98	0.43 ± 0.03	0	1	0.50
F	123.94 ± 2.55	99	200	38.53	12.86 ± 0.60	2	28	10.23

**Table 3:** Descriptive statistics of bacterial and fungal concentrations 2 kilometers away from the landfill sites.

Location/site	Bacteria (ACGIH limit 100 CFU/m^3^)				Fungi (WHO limit 500 CFU/m^3^)			
	Mean ± SE	Min	Max	SD	Mean ± SE	Min	Maxi	SD
A	10.80 ± 2.60	4	16	5.81	0.60 ± 0.40	0	2	0.89
B	3.80 ± 1.02	1	6	2.28	0.40 ± 0.25	0	1	0.55
C	6.00 ± 0.89	6	8	2.00	1.00 ± 0.45	0	2	1.00
D	3.00 ± 0.71	1	5	1.58	0.40 ± 0.25	0	1	0.55
E	3.40 ± 0.51	2	5	1.14	2.60 ± 0.75	1	5	1.67
F	1.80 ± 0.58	1	4	1.30	0.60 ± 0.40	0	2	0.89

**Table 4:** Occurrence of different bacteria species isolated from landfill sites and homes across the wet and dry seasons.

	A				B				C				D				E				F				Total (%)
	Dump site		Homes		Dump site		Homes		Dump site		Homes		Dump site		Homes		Dump site		Homes		Dump site		Homes		
	Dry	Wet	Dry	Wet	Dry	Wet	Dry	Wet	Dry	Wet	Dry	Wet	Dry	Wet	Dry	Wet	Dry	Wet	Dry	Wet	Dry	Wet	Dry	Wet	
*Acinetobacter lwoffii*	10	8	100	30	99	70	80	50	20	8	80	70	60	35	14	10	30	5	13	2	8	2	3	1	808 (6.5)
*Bacillus spp*	11	8	9	5	99	76	91	69	70	60	59	30	90	78	50	20	15	10	8	3	10	6	5	3	885 (7.2)
*Enterobacter aerogenes*	6	3	4	1	90	69	85	61	60	45	39	20	50	39	28	15	25	18	13	9	5	3	2	1	691 (5.6)
*Enterococcus faecalis*	20	16	15	13	8	2	3	1	50	39	28	15	10	8	3	10	11	8	9	5	11	8	9	5	307 (2.5)
*Escherichia coli*	41	38	39	35	15	10	8	3	25	20	18	13	20	16	15	13	70	56	35	65	43	68	44	90	800 (6.5)
*Micrococcus spp*	8	5	6	3	92	65	80	50	35	25	30	20	40	29	28	15	23	18	15	9	8	5	4	2	615 (5.0)
*Morganella morganii*	25	20	18	13	20	16	15	45	40	38	33	50	26	25	23	45	90	78	50	20	15	10	8	3	726 (5.9)
*Proteus mirabilis*	85	40	68	43	70	56	35	65	43	68	44	90	56	55	33	65	50	39	28	15	25	18	13	9	1113 (9.0)
*Proteus vulgaris*	21	18	19	15	109	86	91	79	80	70	69	40	100	98	60	30	25	20	18	13	20	16	15	13	1125 (9.1)
*Pseudomonas aeruginosa*	33	27	34	55	130	116	121	98	101	95	89	70	125	119	90	60	85	40	68	43	70	56	35	27	1787 (14.5)
*Staphylococcus aureus*	41	38	39	35	129	106	101	99	100	90	89	60	120	108	80	50	45	40	38	33	50	26	25	23	1565 (12.7)
*Staphylococcus epidermidis*	45	37	45	36	150	116	121	110	120	99	98	65	128	118	111	80	65	43	68	44	90	56	55	33	1933 (15.6)
Total	346 (2.8)	258 (2.1)	396 (2.3)	284 (2.3)	1011 (8.2)	788 (6.4)	831 (6.7)	730 (5.9)	744 (6.0)	657 (5.3)	676 (5.5)	543 (4.4)	825 (6.7)	728 (5.9)	535 (4.3)	413 (3.3)	534 (4.3)	375 (3.0)	363 (2.9)	261 (2.1)	355 (2.9)	274 (2.2)	218 (1.8)	210 (1.7)	12355 (100)

**Table 5:** Occurrence of different fungi species isolated from landfill sites and homes across the wet and dry seasons.

Fungi	A				B				C				D				E				F				Total (%)
	Dump site		Homes		Dump site		Homes		Dump site		Homes		Dump site		Homes		Dump site		Homes		Dump site		Homes		
	Dry	Wet	Dry	Wet	Dry	Wet	Dry	Wet	Dry	Wet	Dry	Wet	Dry	Wet	Dry	Wet	Dry	Wet	Dry	Wet	Dry	Wet	Dry	Wet	
*Aspergillus niger*	18	7	4	1	0	0	0	0	8	2	1	0	0	0	0	0	5	3	2	1	8	2	1	0	63 (10.5)
*Aspergillus brevipes*	12	5	2	1	4	2	1	0	12	6	2	1	4	2	0	0	9	6	3	1	9	6	3	1	92 (15.3)
*Aspergillus flavus*	0	0	0	0	9	5	2	0	0	0	0	0	8	3	2	1	9	4	2	0	2	0	0	0	47 (7.8)
*Aspergillus parasiticus Speare*	2	0	0	0	7	3	2	1	0	0	0	0	3	0	0	0	7	4	3	0	5	2	2	1	42 (7.0)
*Cladosporium spp*	12	3	1	0	6	10	0	0	18	5	2	2	5	2	0	0	10	5	2	1	12	7	3	1	107 (17.8)
*Penicillium halicum*	5	3	2	0	0	0	0	0	0	0	0	0	4	1	0	0	6	3	2	1	0	0	0	0	27 (4.5)
*Phanerochaete chrysosporium*	7	4	3	0	5	2	2	1	9	6	3	1	9	6	3	1	2	0	0	0	7	3	2	1	77 (12.8)
*Rhizopus stolonifer*	12	6	2	1	4	2	0	0	9	6	3	1	9	6	3	1	9	4	2	0	2	0	0	0	82 (13.6)
*Ulocladium chartarum*	4	2	1	0	12	6	2	1	7	3	2	1	0	0	0	0	7	4	3	0	5	2	2	112.0	65 (10.8)
Total	72 (12.0)	30 (5.0)	15 (2.5)	3 (0.5)	47 (7.8)	30 (5.0)	9 (1.5)	3 (0.5)	63 (10.5)	28 (4.7)	13 (2.2)	6 (1.0)	42 (7.0)	20 (3.3)	8 (1.3)	3 (0.5)	64 (10.5)	33 (5.5)	19 (3.2)	4 (0.7)	50 (8.3)	22 (3.7)	13 (2.2)	5 (0.8)	602 (100)

## Data Availability

The data used to support the findings of this study are included within the article.
